# Seeking and receiving hypertension and diabetes mellitus care in Tanzania

**DOI:** 10.1371/journal.pone.0312258

**Published:** 2024-11-22

**Authors:** Kassimu Tani, Brianna Osetinsky, Grace Mhalu, Sally Mtenga, Günther Fink, Fabrizio Tediosi

**Affiliations:** 1 Ifakara Health Institute, Dar es Salaam, Tanzania; 2 Swiss Tropical and Public Health Institute, Allschwil, Switzerland; 3 University of Basel, Basel, Switzerland; University of Health Sciences Lahore, PAKISTAN

## Abstract

The rapid increase in chronic non-communicable diseases (NCDs) poses a major challenge to already strained health systems in sub-Saharan Africa. This study investigates the factors associated with seeking and receiving NCD services in Tanzania, using a household survey and client exit interview data from Kilombero and Same districts. Both districts are predominantly rural, with one semi-urban area called Ifakara town and Same town. Of the 784 household survey respondents, 317 (40.4%), 37 (4.7%), and 20 (2.5%) were diagnosed with hypertension, diabetes mellitus, and other NCDs, respectively, of whom 69% had sought care in the past six months. After controlling for covariates, those enrolled in the National Health Insurance Fund (NHIF) and those who received a user fees waiver were more likely to use health services. However, even when NCD patients managed to access the care they needed, they were likely to receive incomplete services. The main reason for not receiving all services at the health facility visited on the day of the survey was drug stock-outs. Among health care users, those registered with the improved Community Health Funds (iCHF) were less likely to receive all prescribed services at the health facility visited than uninsured patients. The findings of this study highlight the need to strengthen both primary care and social health protection systems to improve access to needed care for NCD patients.

## Introduction

The burden of chronic non-communicable diseases (NCDs), such as hypertension, diabetes, asthma, chronic lung disease and other cardiovascular diseases (CVD), is increasing rapidly [1–5], in sub-Saharan Africa (SSA). This poses a challenge to the health systems in the region [5–7]. Limited resources are available to provide adequate health services in SSA, resulting in a small proportion of patients accessing care for NCDs [6,7]. In Tanzania, a study conducted in 2013 estimated that 58% of the patients receiving outpatient services at district hospitals, 20% at health centres outpatient clinics and 13% of dispensaries were seeking care for chronic conditions [8].

The quality and availability of NCD services varies geographically [9,10], by facility level and type, and across socioeconomic groups [11,12]. In 2019, the Ministry of Health, in partnership with the Tanzania NCDs Alliance and the Tanzania Diabetes Association, committed to establishing more than 150 NCD clinics across the country [13] to improve service availability and uptake [11,14,15]. This initiative included staff training and the provision of initial NCD diagnostic kits, aiming to strengthen health centres and dispensaries [13], as the management of NCDs is critical at primary care level [16].

More than 50% of the estimated costs of NCD care are directly borne by the patient in the form of out of pocket payments [17]. Health insurance or user fee waivers programs could reduce the financial barriers that prevent people with NCDs from seeking and accessing necessary health services [18–22]. Evidence from a number of studies conducted in sub-Saharan African countries suggests that patients without health insurance are less likely to seek health care in general and are more likely to face catastrophic expenditure on healthcare compared with those who have health insurance or are enrolled in a user fee exemption program [11,23–26].

There are several social health protection schemes in Tanzania that cover payments for health services. The National Health Insurance Fund (NHIF) targets formal sector workers, children under 18 years and students, and is gradually also trying to reach the informal sector through a number of private packages [27]. The improved Community Health Fund (iCHF) is explicitly designed to cover informal sector households. There are also other private insurance schemes that often cover the formal sector [28]. In addition, there are user fee waivers based on age, poverty and certain health conditions [29,30]. However, the coverage of exemptions/waiver and health insurance is low, and there is lack of evidence on their role in facilitating access to health services for people with NCDs [31].

There is still insufficient evidence on the determinants of access to different healthcare providers among people with NCDs living in rural areas. Several studies have highlighted a worrying trend of patient’s often first seeking care at advanced stages of their disease [11,32], coupled with the inadequate readiness of health facilities to provide comprehensive NCD care [12,33,34]. This study therefore endeavours to address the question: What are the factors that influence seeking and receiving NCD care?

This study therefore aims to explore the factors that underpin the seeking and receiving of care. To this end, this study utilized a combination of household and client exit surveys recently conducted in the Kilombero and Same districts of Tanzania. First, it examined the factors associated with access to formal health services by people with hypertension and/or diabetes. Second, it aimed to identify the determinants associated with the completeness of care received for hypertension and diabetes, including the likelihood of promptly receiving all necessary services at the health facility visited. The study also examined the role of health insurance, user fees and exemption schemes in facilitating access to health services for the study population.

## Methods

### Study design

We used data from two cross-sectional surveys, a household survey and a client exit survey at health facilities, conducted between November 2020 and January 2021. The questions in the two surveys were similar, except that the client exit survey included additional questions about the specific health services received on the day of the interview.

### Study settings

The surveys were conducted in Kilombero and Same districts (Table 1). Kilombero is one of the seven districts of Morogoro region in southwestern Tanzania while Same is one of the seven districts of the Kilimanjaro region in the northern part of the country. Both districts are predominantly rural with a single semi-urban area called Ifakara town and Same town, respectively. The majority of villagers in Kilombero and Same are subsistence farmers with some also engaged in livestock rearing and fishing. The Kilombero and Same districts were purposively selected as both have received implementation support from international organisations and non-governmental organisations (NGO) for expanding insurance coverage with both districts being among the first to implement community health funds, exemptions and waivers to improve social health protection and access to health care [21,28,35]. Working in the Kilombero district was additionally benefit due to its longstanding research collaboration with the Ifakara Health Institute (IHI) in Kilombero, where Ifakara serves as the main field centre.

**Table 1:** District summary statistics 2020.

Category	Kilombero District	Same District
Geographical size (square kilometre)	14,918	6,221
Population	558,241	296,287
Wards	26	31
Villages/streets	99	93
Number of health facilities	Dispensaries 61 Health Centre 8 Hospital 2	Dispensaries 62 Health Centre 8 Hospital 2
Health facility per 10,000 population	0.786	0.412

### Household survey

#### Sampling

A multistage clustered sampling design was used in Kilombero and Same districts, where wards (n = 34) of these districts were sampled proportionally to population size with replacement, and villages/streets (n = 52) within the wards were selected by simple random sampling. The survey was part of a larger project investigating health expenditure and use of chronic disease care in Tanzania [36]. We collected data in 26 village clusters per district, randomly sampling the cluster in proportion to the population size of the each ward. Since the towns of Ifakara and Same form their own administrative wards, street names *(Mtaa in Swahili)* rather than villages were randomly selected as clusters. Within each cluster, 15 households were randomly selected from a list of households provided by the village head on the day of the survey. In the urban areas of Same and Ifakara town, where the population size is larger, we allocated 8 sampling clusters. We then generated random geographical starting points and sampled every 3^rd^ household counter-clockwise around the block. A random sample of 780 households was interviewed (Table 2). Further details about the survey can be found elsewhere [36].

**Table 2:** Sample size and sampling strata.

Study Type	Sample Size	Strata
Household Survey	**Kilombero District:**	
	21 villages	4 streets—Ifakara town
	15 households per village/street	Sample of 26 villages/streets town (87 total)
	**Same District:**	
	21 villages	4 streets—Same town
	15 households per village/street	Sample of 26 villages/streets town (91 total)
	**Total: 780 households**	**Total: 52 villages/towns**
Exit Interviews	**Kilombero District:** • 18 health facilities	
	72 per hospital	• 2 hospitals (2 total)
	72 per health centre	• 8 health centres (8 total)
	12 per dispensary	• 8 dispensaries (71 total)
	**Same District:** • 18 health facilities	
	72 per hospital	• 2 hospitals (2 total)
	72 per health centre	• 8 health centres (8 total)
	12 per dispensary	• 8 dispensaries (68 total)
	**Total: 1,632**	**Total: 36 health facilities**

#### Sample size

The sample size was calculated based on a hypertension prevalence of 19.9% (95% confidence interval (CI): 17.1 to 22.9) and a diabetes prevalence of 14.8% (95% CI: 12.4 to 17.6) for adults in rural Tanzania [37]. We also considered the population estimates for Kilombero and Same districts from the 2012 Tanzania census and a benchmark average annual household health expenditure of 30,000 Tanzanian shillings [38]. Using a modified Cochran sample size calculation (power of 0.80, significance level of 0.05) [39–41], we determined that a minimum sample size of 202 households per district was required. However, due to limited information on the seeking and receiving care, we increased the total sample size to 390 households per district.

#### Data collection procedure

During the field research, the research assistant in-charge visited each household and explained the purpose of their visit: to collect information about health care utilization and expenditures within the household. The research assistant then obtained consent from a representative over the age of 18 who would be available in the village on the day of data collection. The representative could be the head of household or another person who could accurately answer questions regarding the household’s healthcare use. Research assistances then interviewed participants to collect information on household demographics and economic characteristics, social health protection, chronic diseases, access to health care, household health expenditures, out-of-pocket health care payments. Research assistances also measured participants’ of blood pressure and random blood glucose.

To ensure accuracy, we used a validated and calibrated digital sphygmomanometer (OMRON M6 Comfort HEM-7321-E) to measure blood pressure and a digital glucometer (On-Call Plus EZ II) to measure blood glucose [36]. Following WHO guidelines for blood pressure screening, each participant was seated and their blood pressure was measured three times, with at least a 5-minute interval between each reading. For the purpose of this study, we considered participants to have elevated blood pressure/hypertension if their mean systolic BP was ≥140 mmHg and/or their mean diastolic BP ≥90 mmHg [42–44]. We calculated the average of the second and third readings to determine the respondent’s blood pressure [45]. Random blood glucose (RBG) was measured using a point-of-care glucometer and a blood sample from the second or third finger. The diagnostic cut-offs for fasting plasma glucose (FPG) were: ≥7.0 mmol/L and for respondents who had eaten at least two hours before testing: ≥11.1 mmol/L [46,47]. If a respondent’s blood pressure or blood glucose exceeded the normal cut-offs, they were informed that their readings were elevated and were advised to contact a healthcare provider at the nearest health facility for further testing.

#### Data management

Data collection from the household survey and the patient exit survey, described in the next section, was conducted over a six-week period from November 2020 to January 2021 by two teams of eight data collectors with backgrounds in medical training and/or public health. One member of each team was a supervisor. Data were collected using the Open Data Kit (ODK) software.

The data collection tools included appropriate skip logic and several validation algorithms, and team supervisors and study authors reviewed the data on a daily basis to identify any errors, inaccuracies, or inconsistencies.

#### Household survey variables

The outcome variable for this study is care-seeking status, dichotomised as ’not seeking care (0)’ and ’seeking care (1)’. A respondent was classified as seeking care if they reported having visited a formal health facility within the previous six months.

Independent variables included social health protection, defined as enrollment in schemes aimed at reducing out-of-pocket payments for formal health services. The area of residence was classified as urban if it was located within the administrative units named streets and if they were grouped together forms a ward, otherwise it was classified as rural if the administrative names were villages and when grouped formed a ward administration unit [48]. The health status of the respondent was categorised according to diagnosis or positive screening for hypertension only, diabetes only, or both hypertension and diabetes, and other NCD conditions. Demographic characteristics such as gender, education level, age, marital status, and occupation were also considered as independent variables.

#### Analysis

We started with descriptive statistics. We distinguished between respondents who reported seeking health care and those who did not, which we used to generate frequency tables. We performed bivariate analysis for each of the explanatory variables for the outcomes of seeking care and not seeking care to assess the significance of the association between seeking care status and each independent variable. We cross-tabulated care-seeking status by each independent variable and presented chi-square p-values.

We used multivariate logistic regression to assess the factors associated with seeking health care in the six months prior to the households’ survey interview. Our focus was whether enrolment in social health protection schemes is associated with obtaining NCD care. Other co-variates of interest were the respondent’s health condition (i.e. not diagnosed/screened positive for hypertension or diabetes, diagnosed/screened positive for hypertension only, or diagnosed/screened positive for hypertension and diabetes), area of residence, gender, education level, age, marital status and occupation.

We drew upon existing literature and knowledge to identify a set of relevant variables before conducting the analysis. We compared the group of respondents who sought care using the chi-square test for univariate analysis. The selection of the variable was guided by univariate logistic regression with a p-value of less than 0.2 and relevant literature in the field to ensure that we were not including unnecessary variables. We used backward elimination to fit the model. Finally, we assessed the goodness-of-fit of the model using the Hosmer-Lemeshow test. This helped us to determine whether the final model was well fitted and accurately described the data [49]. We analysed data using Stata version 16 [50].

### Patient exit survey

#### Sampling technique

Research assistances administered the survey at all hospitals (n = 4), all health centres (n = 16) and a random sample of eight dispensaries in both the Kilombero and Same districts (n = 16). As each of these districts has eight health centres, dispensary sampling matched by randomly selection of one dispensary from each ward containing a health centre. All adult patients aged 18 and above who entered the outpatient clinic on the day of the survey were eligible for recruitment, regardless of whether they were seeking care for NCDs or another condition (Table 2).

This study employed operational random sampling of participants by selecting and recruiting them based on the order in which they entered the consultation room. As demonstrated by Geldsetzer et al. (2018), sampling patients as they enter the consultation room is more efficient and simpler to implement than other random sampling approaches, and it minimises the bias of consultation length associated with sampling patients at the end of consultation [51]. Research assistances interviewed clients after the completion of all services, including the collection of prescribed medications at the facility pharmacy if they were available. Across both districts, we were anticipating a total sample size of 1,632 for the exit survey (Table 2). The final survey implementation resulted in 1748 respondents.

#### Data collection procedure

Hospitals and health centres typically designate one day per week as an ‘NCD day’, or hypertension and diabetes outpatient clinic day, where a medical officer and a trained nurse are assigned to provide outpatient hypertension and diabetes care [12,36]. On these days, patients receive a range of services including medical consultations, nursing care, medication management, health education, and medication refills from the health facility pharmacy. Therefore, we purposively conducted the survey on at least one NCD day in each sampled hospital and health centre to ensure that we reach a sufficient number of participants with NCDs.

#### Client exit survey variables

The outcome variable for this study is access to care, categorised as ’incomplete access (0)’ and ’complete access (1)’. Patients were classified as having complete access if they reported receiving all necessary services at the health facility visited, as prescribed by their health care provider. This included services such as registration, consultation, laboratory tests, prescriptions and all necessary supplies, including medicines. Patients who reported not receiving at least one required service at the health facility on the day they sought care were classified as having incomplete access to services. Independent variables included socio-demographic factors, enrolment in social health protection schemes, reason for seeking care, health facility level and locality. Health facilities were categorised as urban if they were located within administrative designations named streets, and when grouped form a ward, otherwise it was classified as rural if the administrative designations named villages are the one forming a ward administration unit [48].

#### Analysis

We first generated descriptive statistics of the data. We differentiated between patients who received all of their prescribed health services and those who received incomplete or partial access to those health services. We conducted bivariate analysis for each of the explanatory variables for the outcomes of complete and incomplete access to health care to assess the significance of the association between access care status and each independent variable. We cross–tabulated of access to care status by each independent variable and presented Chi-square p-values.

We used multivariate logistic regression to investigate the factors associated with receiving essential health services at the health facility. This model included factors that could influence access to services and the type of services received, including socio-demographics, enrolment in social health protection schemes, reason for seeking care, health facility level and locality.

We drew upon existing literature and knowledge to identify a set of relevant variables before conducting the analysis. We used backward elimination to fit the model. Finally, we assessed the goodness-of-fit of the model using the Hosmer-Lemeshow test. This helped us to determine if the final model was well-fitted and accurately described the data [49]. Data was analysed using Stata version 16.

### Ethics approval and consent to participate

This study was reviewed and approved by the institutional review board of Ifakara Health Institute (IHI) approval number IHI/IRB/No:35–2020 and the Tanzania National Research Coordinating Committee at National Institute for Medical Research (NIMR) approval number NIMR/HQ/R.8a/Vol.IX/3518. All activities has been performed in accordance with IHI and NIMR guidelines and regulations on human research. Informed consent was taken in a series of stages. The ministry of local government was informed to provide permission to conduct survey at districts. The districts level provided permission to enter into health facility and community for survey by providing letter to introduce the research team to the health facility and community leaders. At health facility, we obtained written informed consent from all study participants accepted to be interviewed. At community, we obtained informed consent from head of household and written consent from every participant to collect data as well as to screen for hypertension and diabetes. Participants had the opportunity to opt out of the hypertension and diabetes screening and continued to participate. We assured confidentiality and anonymity in the process of collection, cleaning, analysis, and reporting of the study findings.

## Results

### Descriptive statistics

Table 3 shows the descriptive statistics of the sample included in the two surveys. Out of 784 household survey respondents, 317 (40.4%), 37 (4.7%), and 20 (2.5%) were diagnosed to have hypertension, diabetes mellitus, and other NCDs, respectively. About 24% of the respondents reported to have an NCD diagnosis prior to the survey and 69% sought care in the last six months. Of the respondents screened for hypertension on the day of survey, 36.6% were found to have high blood pressure according to the survey’s measurements. 67% had at least basic primary education and 71% were engaged in farming activities. Of female respondents, 28.8% sought care compared with 22.3% of males, and 32.1% of the urban respondents sought care compared to 24.1% in rural areas. We observed a significantly higher percentage of insured or fee-exempt participants using health services (ranging from 30% to 64%), relative to 22% of those who were not covered by any social health protection scheme. Furthermore, among those who were diagnosed with hypertension and diabetes, 41% and 88% sought care in the last six months, respectively.

**Table 3:** Bivariate analysis of seeking and receiving care by demographics and NCD diagnosis of the study participants.

	Household survey			Health facility patient survey		
Characteristic	Total n = 784, n (%)	Bivariate analysis			Bivariate analysis	
		Seeking care n = 208, n (%)	p-value	Total n = 1748, n (%)	Complete access n = 1131, n(%)	p-value
**Residence/locality**						
Urban	240 (30.6)	77 (32.1)	0.02*	1265 (72.4)	823 (65.1)	0.61
Rural	544 (69.4)	131 (24.1)		483 (27.6)	308 (63.8)	
**Gender**						
Female	511 (65.2)	147 (28.8)	0.05	1114 (63.7)	701 (62.9)	0.04*
Male	273 (34.8)	61 (22.3)		634 (36.3)	430 (67.8)	
**Age Categories**						
< = 35	200(25.5)	43 (21.5)	0.00***	662 (37.9)	439 (66.3)	0.35
36–55	346 (44.1)	75 (21.7)		569 (32.5)	370 (65.0)	
> = 56	238 (30.4)	90 (37.8)		517 (29.6)	322 (62.3)	
**Education Level**						
No education	150 (19.1)	33 (22.0)	0.38	299 (17.1)	190 (63.5)	0.32
Primary completed	522 (66.6)	144 (27.6)		992 (56.8)	632 (63.7)	
Secondary and above	112 (14.3)	31 (27.7)		457 (26.1)	309 (67.6)	
**Occupation**						
Formal employed	65 (8.3)	21 (32.3)	0.00***	418 (23.9)	301 (72.0)	0.01**
Farmers	559 (71.3)	131 (23.4)		942 (53.9)	587 (62.3)	
Self-employed	140 (17.7)	42 (30.0)		307 (17.6)	192 (62.5)	
Retired	20 (2.6)	14 (70.0)		81 (4.6)	51 (63.0)	
**Marital status**						
Never married	53 (6.8)	9 (17.0)	0.07	270 (15.4)	175 (64.8)	0.37
Married	583 (74.3)	151 (25.9)		1179 (67.5)	773 (65.6)	
Separated/divorce	148 (18.9)	48 (32.4)		299 (17.1)	183 (61.2)	
**Modal of social health protection**						
No SHP	634 (80.9)	142 (22.4)	0.00***	924 (52.9)	612 (66.2)	0.01**
iCHF	33 (4.2)	10 (30.3)		216 (12.4)	118 (54.6)	
NHIF	74 (9.4)	32 (43.2)		438 (25.1)	302 (68.9)	
Private insurance	11 (1.4)	7 (63.6)		35 (2.0)	21 (60.0)	
Exemption	32 (4.1)	17 (53.1)		135 (7.7)	78 (57.8)	
**NCD Diagnosed (prior)**						
No NCDs	597 (76.4)	80 (13.4)	0.00***	1214 (69.5)	802 (66.1)	0.07
NCDs	187 (23.6)	128 (69.2)		534 (31.5)	329 (61.6)	
No NCDs	764 (97.5)	194 (25.4)	0.00***	1656 (94.7)	1079 (65.2)	
Other NCDs (not HTN/DM)	20 (2.5)	14 (70.0)		92 (5.3)	52 (56.5)	0.09
**HTN (Screened or reported)**						
No	467 (59.6)	78 (16.7)	0.00***	1317 (75.3)	874 (66.4)	0.67
Yes	317 (40.4)	130 (41.0)		431 (24.7)	257 (59.6)	
**DM (Screened or reported)**						
No	747 (95.3)	176 (23.6)	0.00***	1621 (92.7)	1051 (64.8)	0.01**
Yes	37 (4.7)	32(88.5)		127 (7.3)	80 (63.0)	
HTN Screened^#^ (survey)						
No	491 (63.7)	111 (22.6)	0.00***	-	-	-
Yes	280 (36.3)	94 (33.6)		-	-	-
**Facility level**						
Dispensary	-	-	-	166 (9.5)	100 (60.2)	0.00***
Health centre	-	-	-	1173 (67.1)	734 (62.6)	
Hospital	-	-	-	409 (23.4)	297 (72.6)	

* p<0.05

** p<0.01

*** p<0.001.

^#^13 Patients did not consent to take blood pressure metrics.

HTN (prior & survey)–Hypertension screened on the day of survey and ever told or prior informed to have condition.

DM (prior & survey)–Diabetes screened on the day of survey and ever told or prior informed to have condition.

The majority of patients interviewed at health facilities were female (64%), and 30% were older than 55 years of age. Respondents largely had basic primary education (57%) and were engaged in farming activities (54%). About 25% of patients were enrolled in the NHIF, 12% in the iCHF, and 8% were granted a user fees waiver and 2% had a private health insurance. Of patients who were formally employed 72% received all required services in the facility visited compared to around 62% - 63% of the other occupational groups. Almost 69% of patients registered with the NHIF received all services in the health facility visited compared to a slightly lower percentage for patients that did not have any insurance (66.2%), while only 54.6% of those registered with iCHF, and 60% of patients with a private insurance received all services. There were some differences in access to required services across the health facility levels, as 73% of patients who visited a hospital received all required services compared to 63% of those that visited a health centre and 60% who visited dispensaries.

Most of the patients reported that the main reason for not receiving all required services in the facility on the day of survey was medicine stock-outs (96%). The out of stock of the medicines prescribed on the day of survey ranged from 10% to 66% (S1 and S2 Tables).

### Logistic regression results on factors associated to seeking health care

Table 4 shows the results of logistic regressions investigating the factors associated with seeking care from the household survey. The individuals enrolled with a social health protection schemes were more likely to have sought health services than the rest of the sample. However, when controlling for other covariates the association held only for patients with a private insurance and for those who were granted a user fee waiver. Education (AOR = 1.99; 95% CI = 1.17–3.38; p = 0.01) and gender (AOR = 0.56; 95% CI = 0.36–0.82; p = 0.01) of the individual were associated with seeking care. The multivariate logistic regression showed that people aged between 36 and 55 years were less likely to seek care compared to the younger people while the association did not hold for older age groups. As expected, people with a diagnosed NCD were more likely to seek care. However, individuals with hypertension only were 3 times more likely to seek care (AOR = 3.09; 95% CI = 2.07–4.61; p = 0.00). Moreover, those with both hypertension and diabetes were 29 times more likely to seek health care compared to those not screened positive for hypertension or diabetes (AOR = 28.92; 95% CI = 10.09–82.84; p = 0.00).

**Table 4:** Univariate and multivariate logistic regression to predict the role of social health protection on seeking health care (households’ survey).

	Univariate		Multivariate	
Variable	OR (95% CI)	p-value	AOR (95% CI)	p-value
**Modal of social health protection**				
no SHP	1.00	-	1.00	-
iCHF	1.36 (0.61–2.99)	0.45	1.02 (0.38–2.60)	0.99
NHIF	2.68 (1.62–4.45)	0.00***	1.73 (0.93–3.25)	0.09
Private insurance	6.93 (1.71–28.06)	0.01**	7.51 (1.56–36.06)	0.01**
Waiver	3.92 (1.91–8.06)	0.00***	3.63 (1.56–8.43)	0.00***
**Gender**				
Female	1.00	-	1.00	-
Male	0.71 (0.51–1.01)	0.06	0.56 (0.36–0.87)	0.01**
**Age**				
Below 35	1.00	-	1.00	-
36–55	1.01 (0.66–1.54)	0.96	0.63 (0.37–1.05)	0.08
Above 55	2.19 (1.42–3.36)	0.00***	0.88 (0.48–1.61)	0.67
**Education level**				
No education	1.00	-	1.00	-
Completed primary	1.43 (0.92–2.22)	0.11	1.99 (1.17–3.38)	0.01**
Secondary and above	1.48 (0.83–2.63)	0.18	1.96 (0.91–4.27)	0.09
**Occupation**				
**Employed (public/private)**	1.00	-	1.00	-
Farmer	0.66 (0.38–1.17)	0.15	0.68 (0.34–1.37)	0.29
Self employed	0.94 (0.49–1.79)	0.85	0.96 (0.45–2.05)	0.93
Retired	7.69 (2.24–26.35)	0.00***	5.31 (1.27–22.19)	0.02*
**Marital**				
Never married	1.00	-	1.00	-
Married	1.71 (0.81–3.57)	0.16	2.78 (1.15–6.71)	0.02*
Separated/divorce	2.27 (1.02–5.04)	0.04*	2.22 (0.83–5.88)	0.11
**Area of residence/locality**				
Urban	1.00	-	1.00	-
Rural	0.67 (0.47–0.93)	0.02*	0.66 (0.45–0.98)	0.04*
**Health condition**				
Not screened positive for Hypertension or Diabetes	1.00	-	1.00	-
Hypertension	3.09 (2.18–4.39)	0.00***	3.09 (2.07–4.61)	0.00***
Comorbid (hypertension and diabetes)	27.32 (10.12–73.75)	0.00***	28.92 (10.09–82.84)	0.00***

* p<0.05

** p<0.01

*** p<0.001.

OR = Odd ratio.

AOR = Adjusted odd ratio.

CI = Confidence interval.

### Logistic regression results on completeness of services received at the health facility visited

Table 5 shows the results of the logistic regressions investigating the factors associated with receiving all required health services at health facilities from the client exit interviews. After controlling for the other covariates, patients enrolled in the iCHF scheme (AOR = 0.65; 95% CI = 0.47–0.90; p = 0.01) were 35% less likely to receive all of their prescribed health services. Participant’s occupation was weakly associated with receiving health services; farmers (AOR = 0.69; 95% CI = 0.51–0.93; p = 0.02) and self-employed (AOR = 0.70; 95% CI = 0.50–0.97; p = 0.04) participants were less likely to receive all prescribed services in the facility compared to participants employed in the formal sector. Patients that received services at hospital (AOR = 1.76; 95% CI = 1.12–2.76; p = 0.02) were 1.76 times more likely to receive all services in the facility compared to those who sought care in dispensaries. Although the results were not statistically significant, we observed that patients with non-communicable diseases (NCDs) were 20% less likely (with an adjusted odds ratio of 0.80) to receive all healthcare services at health facilities compared to patients with other conditions.

**Table 5:** Univariate and multivariate logistic regression to predict the role of social health protection on receiving full health services (health facility patient survey).

	Univariate		Multivariate	
Variable	OR (95% CI)	p-value	AOR (95% CI)	p-value
**Modal of social health protection**				
no SHP	1.00	-	1.00	-
iCHF	0.61 (0.46–0.83)	0.00***	0.65 (0.47–0.90)	0.01**
NHIF	1.14 (0.89–1.45)	0.29	1.12 (0.84–1.51)	0.41
Private insurance	0.69 (0.32–1.52)	0.37	0.76 (0.33–1.73)	0.53
Waiver	0.69 (0.48–1.01)	0.06	0.77 (0.51–1.15)	0.21
**Gender**				
Male	1.00	-	1.00	-
Female	1.24 (1.01–1.52)	0.04*	1.17 (0.94–1.47)	0.15
**Age (Years)**				
Below 35	1.00	-	1.00	-
36–55	0.93 (0.74–1.18)	0.59	0.97 (0.74–1.28)	0.86
Above 55	0.84 (0.66–1.08)	0.18	0.90 (0.63–1.29)	0.58
**Education level**				
No education	1.00	-	1.00	-
Completed primary	0.97 (0.74–1.28)	0.84	0.84 (0.62–1.13)	0.25
Secondary and above	1.17 (0.85–1.59)	0.33	0.85 (0.58–1.23)	0.39
**Occupation**				
Employed (public/private)	1.00	-	1.00	-
Farmer	0.64 (0.49–0.83)	0.00***	0.69 (0.51–0.93)	0.02*
Self employed	0.64 (0.46–0.87)	0.01**	0.70 (0.50–0.97)	0.04*
Retired	0.64 (0.38–1.06)	0.08	0.63 (0.36–1.10)	0.11
**Marital**				
Never married	1.00	-	1.00	-
Married	1.04 (0.78–1.37)	0.78	1.21 (0.88–1.67)	0.24
Separated/divorce	0.88 (0.62–1.24)	0.47	1.18 (0.77–1.81)	0.44
**Area of residence/locality**				
Urban	1.00	-	1.00	-
Rural	0.94 (0.75–1.18)	0.61	1.09 (0.83–1.41)	0.51
**Health condition reported**				
Not NCDs	1.00	-	1.00	-
NCDs	0.82 (0.66–1.02)	0.08	0.80 (0.62–1.03)	0.09
**Facility level**				
Dispensary	1.00	-	1.00	-
Health centre	1.10 (0.78–1.54)	0.57	1.21 (0.83–1.75)	0.33
Hospital	1.73 (1.18–2.53)	0.01**	1.76 (1.12–2.76)	0.02*

* p<0.05

** p<0.01

*** p<0.001.

OR = Odd ratio.

AOR = Adjusted odd ratio.

CI = Confidence interval.

## Discussion

In this study, we used a household survey recently conducted in Tanzania to investigate the factors associated with access to formal health services for people with hypertension and diabetes. We also utilized client exit interviews from the same districts to identify barriers to accessing necessary services, and to assess factors affecting the likelihood of receiving all required services in a timely manner at the health facilities visited. We also examined role of health insurance, user fees, and exemption schemes in facilitating access to health services.

Overall, the proportion of respondents seeking healthcare was low compared to similar studies conducted in other countries, such as Burkina Faso and Malawi [52–55]. A higher prevalence of self-medication [53,56–59] and more limited access to health services [11] could explain the lower prevalence of health seeking in our sample. Our study also revealed that enrolment in a private insurance scheme or benefiting from a user fee waiver were associated with an increased probability of seeking healthcare services. These results are consistent with the findings of previous studies conducted in Kenya, Ghana, Malawi, and southern regions of Tanzania [11,53,54,60–62].

As expected, patients diagnosed with hypertension and diabetes were more likely to seek healthcare services than those with other conditions. Given the need for regular treatment, these participants were more likely to have required care during the six months preceding the survey [33,61,63]. However, despite seeking care, patients with hypertension and diabetes were less likely to receive all needed health services at the health facility compared to patient with other conditions. This finding is likely due to the nature of the services required for hypertension and diabetes, as well as the high frequency of medicine stock outs [64,65]. Enrollment in the iCHF was negatively associated with receiving all necessary health services at the health facilities visited. This could be due to implementation challenges of the iCHF scheme, particularly in ensuring timely access to quality services in rural areas. This finding may be related to the fact that due to more limited reimbursement under the iCHF scheme, providers seem to prioritise out-of-pocket payments over iCHF-insured patients, as documented in recent studies [19,66,67]. However, although the results were not statistically significant (AOR = 1.12, p-values = 0.41), NHIF membership was associated with a higher likelihood of receiving all prescribed health services at health facilities, possibly due to the broader network of health facilities and the comprehensive package of services offered by the NHIF. These findings are consistent with previous studies conducted in Kenya and Ghana, which indicate that most patients enrolled in national compulsory insurance schemes receive the necessary health care services [61,62]. In addition, other studies have shown that patients diagnosed with hypertension and diabetes enrolled in a national health insurance scheme have better engagement in care and adherence to follow-up appointments [11,36,53,68], due to reduced financial barriers to care [17].

Patients with hypertension and diabetes had higher probability of receiving all needed services when seeking care in hospitals relative to health centres and dispensaries, which is consistent with the results of other studies [12,69,70]. However, in Tanzania, despite efforts to improve NCD care at the primary care levels, more resources have been invested in strengthening hospitals, while dispensaries and health centres still struggle with inadequate human resources, training and the poor availability of first-line drugs for NCDs [8]. This highlights the need for investments to strengthen primary care at dispensary and health centre levels in rural Tanzania, including reframing policies guiding NCD care at those levels [13,26,71].

The results of our study also suggest that seeking care for hypertension and diabetes did not vary significantly across socio-economic groups, although there were differences across occupational groups. Those employed in the formal sector were more likely to receive all services, suggesting that improvements in health systems would benefit all people in these settings, regardless of their socio-economic status. To achieve this, efforts should be made to expand enrolment in the NHIF to cover both formal and informal sector workers. The higher likelihood of receiving all needed services among those in the formal sector may be related to the lower health literacy of certain occupational groups and their ability to engage with healthcare providers. Therefore, efforts are needed to support informal sector populations and to increase the responsiveness of healthcare providers [61,72–74].

Patients living in rural areas were found to be less likely to seek care, which may be due to the longer travel time and higher costs associated with accessing health facilities, as reported in previous studies [75,76]. However, when rural patients do seek care, they are more likely to receive all the services needed at the health facility, which may indicate that they attend the health facility when they are reasonably confident that, services and medicines are available or when their health condition has reached a critical point.

The results of our study are consistent with those of previous research, which have shown medicine stock outs are major reasons why patients do not receive all necessary services [12,77,78]. Therefore, it is crucial to ensure that medicines are consistently available by improving the medicine procurement and supply chain. Health facilities should submit requests to the Medical Store Department and engage with authorized private medicine vendors to address medicine shortages [79–81]. This would help improve medicine availability and ensure that patients receive the care they need.

The findings of this study should be interpreted in light of at least five sets of limitations. First, the household survey had a short 6-month period for recalling healthcare seeking behaviour, which may limit the detection of infrequent events such as hospitalisations. Second, there might be an overrepresentation of people with hypertension and diabetes mellitus, as the survey included hypertension and diabetes screening, which may have increased the number of households volunteered respondents with chances of having NCDs. Third, point-of-care fasting glucose measurement is not 100% accurate and patients with diabetes may have been missed. This could underestimate the prevalence of diabetes in the sample. Fourth, the definition of receiving all prescribed health services in this study was limited to a list of self-declared services received by respondents. Lastly, there could be a potential bias if the same respondents happened to have been interviewed in the community during the household survey and in the health facility during the client exit survey.

Despite these limitations, to our knowledge, this is the first study to analyse household and client exit data to measure health-seeking behaviour and care received by patients with hypertension and diabetes in Tanzania. The study design, explored the same community at both the household and health facility levels, demonstrated how payment models facilitate health services. Understanding the gap between seeking and receiving care could help in designing policies to improve community awareness and encourage enrollment in social health protection schemes, addressing barriers to accessing hypertension and diabetes services. These findings have the potential to inform policy-relevant development for social health protection systems in Tanzania and similar low-income countries.

## Conclusion

The findings of this study point to significant challenges in seeking and receiving healthcare for diabetes and hypertension in Tanzania. Individuals with hypertension or diabetes were more likely to have sought care in the past six months than individuals without these conditions. However, once they accessed health services, they often received incomplete care, particularly at the dispensary level, due to medicine stock-outs. Patients employed in the formal sector and enrolled in social health protection schemes other than iCHF were more likely to receive all the services they needed. These findings highlight the need to strengthen primary care, improve the medicine supply chain, and make social protection schemes more inclusive to improve access to necessary services for non-communicable diseases.

## Supporting information

S1 Table Reason for not accessing full-needed health care: By health facility level and social health protection/funding sources. (DOCX)

S2 Table Medicine stock-out by patient prescription on the day of survey. (DOCX)

## Abbreviations

NCDs: Chronic non-communicable diseases
CVD: Cardiovascular diseases
SSA: sub-Saharan Africa
NHIF: National Health Insurance Fund
iCHFs: improved Community Health Funds
RBG: random blood glucose
PO-RALG: President office, ministry of regional and local government administration
SNF: Swiss National Science Foundation
ODK: the Open Data Kit
