# Supplementary material for: Seeking and receiving hypertension and diabetes mellitus care in Tanzania
Source: PLoS One. 2024 Nov 22;19(11):e0312258. doi: 10.1371/journal.pone.0312258 (PMC11584143; doi:10.1371/journal.pone.0312258)
Supplement: S1 Table — (DOCX) [file pone.0312258.s001.docx]

**S1 Table A: Reason for not accessing full-needed health care: by health facility level and social health protection/funding sources**

| **Health protection scheme** | **NCDs** | | | | **Not NCDs** | | | |
| --- | --- | --- | --- | --- | --- | --- | --- | --- |
|  | **Clinic pharmacy was out of stock n (%)** | **Proportional of stock out** | | | **Clinic pharmacy was out of stock n (%)** | **Proportional of stock out** | | |
|  |  | **Hosp (%)** | **HC (%)** | **Disp (%)** |  | **Hosp (%)** | **HC (%)** | **Disp (%)** |
| No SHP | 39 (88.6) | 7.7 | 71.8 | 20.5 | 151 (94.4) | 8.6 | 78.8 | 12.6 |
| iCHF | 22 (95.6) | 4.5 | 90.9 | 4.5 | 57 (98.3) | 8.8 | 86 | 5.3 |
| NHIF | 61 (100) | 68.9 | 27.9 | 3.3 | 51 (98.1) | 15.7 | 70.6 | 13.7 |
| Private  insurance | 7 (100) | 14.3 | 85.7 | 0 | 6 (100) | 0 | 100 | 0 |
| Waiver | 31 (100) | 12.9 | 87.1 | 0 | 20 (95.2) | 0 | 90 | 10 |
| **Total** | **160 (96.4)** | **31.9** | **61.3** | **6.9** | **285 (95.7)** | **9.1** | **80** | **10.9** |

The table above indicate the reason lead to not accessing the required services and the proportion of 579 the patients reported by level of health facilities (Hospitals, Health centres and Dispensaries) and 580 modality of financing health services. Clinic pharmacy out of stock, pronounced as an outstanding 581 reason for partial access to required health care.
