# Supplementary material for: Seeking and receiving hypertension and diabetes mellitus care in Tanzania
Source: PLoS One. 2024 Nov 22;19(11):e0312258. doi: 10.1371/journal.pone.0312258 (PMC11584143; doi:10.1371/journal.pone.0312258)
Supplement: S2 Table — (DOCX) [file pone.0312258.s002.docx]

**S2 Table B: Medicine stock-out by patient prescription on the day of survey**

|  | **Total prescription** | **Total out of stock** | **Proportional of stockout** |
| --- | --- | --- | --- |
| **Diabetes** | | | |
| 1 Metformin | 73 | 21 | 28.8 |
| 2 Glibendinide | 53 | 19 | 35.8 |
| 3 Ligaba | 23 | 10 | 43.5 |
| 4 Insulin injection | 3 | 1 | 33.3 |
|  |  |  |  |
| **Hypertension** | | | |
| 5 Furosemide | 85 | 18 | 21.2 |
| 6 Nifedipine | 105 | 37 | 35.2 |
| 7 Captopril | 68 | 24 | 35.3 |
| 8 Propranolol | 5 | 1 | 20.0 |
| 9 Atenolol | 22 | 8 | 36.4 |
| 10 Amilodipine | 86 | 22 | 25.6 |
| 11 Carvedilol | 25 | 11 | 44.0 |
| 12 Telmisatan | 17 | 10 | 58.8 |
| 13 Spironolactone | 5 | 2 | 40.0 |
| 14 Losartan | 48 | 14 | 29.2 |
| 15 Atorvastatin | 10 | 4 | 40.0 |
| 16 Digoxin | 9 | 1 | 11.1 |
| 17 Clopidogrel | 15 | 7 | 46.7 |
| 18 Aprinox | 27 | 12 | 44.4 |
| 19 Loprin | 13 | 3 | 23.1 |
|  |  |  |  |
| **Anti Malaria** | | | |
| 20 ALU | 88 | 14 | 15.9 |
| 21 Metakefin | 8 | 2 | 25.0 |
| 22 Malafin | 4 | 1 | 25.0 |
| 23 Quinine | 8 | 1 | 12.5 |
| 24 Duo cotexcin | 3 | 1 | 33.3 |
|  |  |  |  |
| **Pain killer** | | | |
| 25 Paracetamol | 479 | 159 | 33.2 |
| 26 declofenac | 1 |  | 0.0 |
| 27 Panadol | 135 | 44 | 32.6 |
| 28 Dicloper | 15 | 11 | 73.3 |
| 29 Diclofenac injection | 118 | 26 | 22.0 |
| 30 Diclofenac gel | 6 |  | 0.0 |
| 31 Aspirin | 43 | 21 | 48.8 |
| 32 Brufen tabs | 58 | 6 | 10.3 |
|  |  |  |  |
| **Antibiotic and others medicine** | | | |
| 33 Cotrimazole caps | 151 | 46 | 30.5 |
| 34 Clotrimazole cream | 33 | 9 | 27.3 |
| 35 Metronidazole |  |  |  |
| 36 Omeprazole | 186 | 46 | 24.7 |
| 39 Tramadol | 24 | 5 | 20.8 |
| 40 Candirstat cream | 2 |  | 0.0 |
| 41 Fluconazole | 31 | 10 | 32.3 |
| 42 Whitefield | 7 | 3 | 42.9 |
| 43 Metronidazole | 16 | 4 | 25.0 |
| 44 Albendazole | 50 | 14 | 28.0 |
| 45 Tinidazole | 18 | 5 | 27.8 |
| 46 Vitamin B complex | 91 | 33 | 36.3 |
| 47 Piriton | 17 | 9 | 52.9 |
| 48 ORS | 39 | 9 | 23.1 |
| 49 Neurobin | 21 | 16 | 76.2 |
| 50 Meloxicam | 23 | 11 | 47.8 |
| 51 Magnesium | 22 | 3 | 13.6 |
| 52 Koflyin | 25 | 8 | 32.0 |
| 53 Zentuss cough | 25 | 8 | 32.0 |
| 54 Sedition cough | 38 | 7 | 18.4 |
| 55 ampicloxine | 13 | 3 | 23.1 |
| 56 Ampiclox caps | 110 | 26 | 23.6 |
| 57 Tretacycline | 4 | 1 | 25.0 |
| 59 Seprine tabs. | 41 | 16 | 39.0 |
| 60 Cetrizine | 63 | 18 | 28.6 |
| 61 Salbutamol | 16 | 4 | 25.0 |
| 62 Pyrazinamide | 3 |  | 0.0 |
| 63 Relcer gel | 11 | 2 | 18.2 |
| 64 Prednisolone Eye Drops | 76 | 26 | 34.2 |
| 65 Powersef injection | 9 | 6 | 66.7 |
| 66 Ampicilline | 277 | 89 | 32.1 |
| 67 ART | 15 | 1 | 6.7 |
| 68 Lamivudine | 1 |  | 0.0 |
| 69 Azithromcycin | 111 | 29 | 26.1 |
| 70 Ciproflaxin | 129 | 39 | 30.2 |
| 71 Bendafluazide | 3 | 1 | 33.3 |
| 72 Betamethasone cream | 12 | 4 | 33.3 |
| 73 Ceftriaxone | 20 | 4 | 20.0 |
| 74 Cetrinaxone injection | 3 |  | 0.0 |
| 75 Cephalexin capsules | 6 | 2 | 33.3 |
| 76 Dexamethasone | 12 | 2 | 16.7 |
| 77 Doxcyline | 61 | 25 | 41.0 |
| 78 Ferrous sulphate | 14 | 3 | 21.4 |
| 79 Flagilly | 21 | 7 | 33.3 |
| 80 Gentamaycine | 16 | 4 | 25.0 |
| 81 Hydrocotisone | 20 | 8 | 40.0 |
| 82 Hyosciline | 16 | 2 | 12.5 |
| 83 Pantoprazole | 8 | 2 | 25.0 |
| 84 Ecoflox-500 | 5 |  | 0.0 |
| 85 Chlorophemine | 6 | 3 | 50.0 |
| 86 Diazepam | 5 | 3 | 60.0 |
| 87 Diabines | 3 | 1 | 33.3 |
| 89 Depin retard | 3 |  | 0.0 |
| 90 Ant-acid | 7 | 2 | 28.6 |
| 91 Adderall | 1 |  | 0.0 |
| 92 Folic acid | 3 | 2 | 66.7 |
| **Total** | **3601** | **1092** | **30.3** |
